# Menstrual Blood-Derived Endometrial Stem Cells’ Impact for the Treatment Perspective of Female Infertility

**DOI:** 10.3390/ijms22136774

**Published:** 2021-06-24

**Authors:** Giedrė Skliutė, Raminta Baušytė, Veronika Borutinskaitė, Giedrė Valiulienė, Algirdas Kaupinis, Mindaugas Valius, Diana Ramašauskaitė, Rūta Navakauskienė

**Affiliations:** 1 Department of Molecular Cell Biology, Institute of Biochemistry, Life Sciences Center, Vilnius University, Saulėtekio av. 7, LT-10257 Vilnius, Lithuania; giedre.skliute@gmc.vu.lt (G.S.); raminta.bausyte@gmc.vu.lt (R.B.); veronika.borutinskaite@bchi.vu.lt (V.B.); giedre.valiuliene@bchi.vu.lt (G.V.); 2 Centre of Obstetrics and Gynaecology of the Institute of Clinical Medicine, Faculty of Medicine, Vilnius University, Santariškių St., LT-08661 Vilnius, Lithuania; diana.ramasauskaite@santa.lt; 3 Proteomic Center, Institute of Biochemistry, Life Sciences Center, Vilnius University, Saulėtekio av. 7, LT-10257 Vilnius, Lithuania; algirdas.kaupinis@gf.vu.lt (A.K.); mindaugas.valius@bchi.vu.lt (M.V.)

**Keywords:** menstrual blood stem cells, infertility, reproductive system diseases, decidualization

## Abstract

When looking for the causes and treatments of infertility, much attention is paid to one of the reproductive tissues—the endometrium. Therefore, endometrial stem cells are an attractive target for infertility studies in women of unexplained origin. Menstrual blood stem cells (MenSCs) are morphologically and functionally similar to cells derived directly from the endometrium; with dual expression of mesenchymal and embryonic cell markers, they proliferate and regenerate better than bone marrow mesenchymal stem cells. In addition, menstrual blood stem cells are extracted in a non-invasive and painless manner. In our study, we analyzed the characteristics and the potential for decidualization of menstrual blood stem cells isolated from healthy volunteers and women diagnosed with infertility. We demonstrated that MenSCs express CD44, CD166, CD16, CD15, BMSC, CD56, CD13 and HLA-ABC surface markers, have proliferative properties, and after induction of menstrual stem cell differentiation into epithelial direction, expression of genes related to decidualization (*PRL*, *ESR*, *IGFBP* and *FOXO1*) and angiogenesis (*HIF1*, *VEGFR2* and *VEGFR3*) increased. Additionally, the p53, p21, H3K27me3 and HyperAcH4 proteins’ expression increased during MenSCs decidualization, they secrete proteins that are involved in the regulation of the actin cytoskeleton, estrogen and relaxin signaling pathways and the management of inflammatory processes. Our findings reveal the potential use of MenSCs for the treatment of reproductive disorders.

## 1. Introduction

Infertility is a disease of the reproductive system. According to statistics, more than 186 million people suffer from infertility, accounting for 8–12% of couples of reproductive age worldwide [1]. The infertility of unexplained origin account for around 10–20% of all fertility problems.

When looking for the causes of fertility problems and treatments, which are used during infertility treatment cycle, much attention is paid to one of the reproductive tissues—the endometrium. The endometrium is a hormone-dependent, cyclically changing, regenerating tissue whose stem cells play a particularly important role in a woman’s reproductive life. Studies show that endometrial dysfunction can lead to failed embryo implantation and early loss of pregnancy, making endometrial stem cell changes an attractive target for infertility studies with unexplained origin [2]. However, endometrial stem cells can only be taken invasively by endometrial scratching, so alternatives to this type of stem cell have been sought.

Studies have shown that menstrual blood could also be a source of stem cells. Experimental studies in animal models have shown successful treatment of stroke, colitis, limb ischemia, coronary heart disease and Duchenne muscle atrophy using menstrual blood stem cells [3]. Menstrual blood is a new and interesting source of MenSCs for researchers because these cells have advantages over stem cells obtained from other sources [4]. Back in 2007, it was shown that MenSCs can even proliferate twice as fast as other mesenchymal stem cells. Proper rate of proliferation is essential for clinical application of cells because cell therapy depends on the number of cells, preferably when many stem cells can be obtained (or propagated) from the same source and are as early in the passage as possible, the process is non-invasive and does not face ethical issues [5]. Because MenSCs are of endometrial origin, attempts are being made to treat conditions associated with female infertility. Nikoo and the group (2014) found that MenSCs are very important for endometrial function by comparing morphology, CD marker expression, cell proliferation, adhesion and some immunomodulatory molecules among women with endometriosis and those without endometriosis [6,7]. Zheng et al. (2018) were the first to show that MenSCs could be a desirable medical tool for reproductive systems cells tissue repair and regeneration [8]. In this study, MenSCs differentiated into endometrial cells in vitro and rebuilt endometrial tissue in NOD-SCID mice after administering estrogen and progesterone in vivo. Additionally, due to results that cloning efficiency and OCT-4 expression of MenSCs from patients with intrauterine adhesions were lower compared with those from healthy women, this study revealed new possibilities to use MenSCs for the treatment of intrauterine adhesions, which remains one of the most challenging fertility problems until now [8]. Moreover, MenSCs are shown to exert a vascular remodeling role and an immunoregulatory role by inhibiting the expression of proinflammatory factors and promoting so-called T helper type 2 (Th2) cytokines such as interleukin-4 (IL-4), which could play a critical role in the embryo implantation process and successful pregnancy development [3,9,10,11].

In our study, we analyzed the characteristics and the potential for decidualization of menstrual blood stem cells isolated from healthy volunteers and women diagnosed with infertility. We have shown that the most analyzed gene expression did not differ in MenSCs of healthy volunteers and females with diagnosed infertility except some epigenetic and stemness gene expression was lower in patients with diagnosed infertility. Therefore, we suggest that MenSCs could be a promising therapeutic source as an auxiliary tool for the females with diagnosed unexplained infertility treatment.

## 2. Results

### 2.1. Characterization of MenSCs Isolated from Healthy vs. Infertile Female

In this study, we characterized MenSCs isolated from healthy volunteers and patients diagnosed with infertility of unexplained origin. MenSCs were cultivated in DMEM/F12 medium as described in the Materials and Methods section. Examination of MenSCs from healthy volunteers showed that cell doubling time was around 3.4 days, while in patients with pathology, it was approx. 3.2 days. The nearly identical proliferation rates of MenSCs from healthy volunteers and patients with unexplained infertility suggest that the rate of proliferation was not affected by the pathology but rather was determined by the individual biological characteristics of each patient.

**Figure 1:**
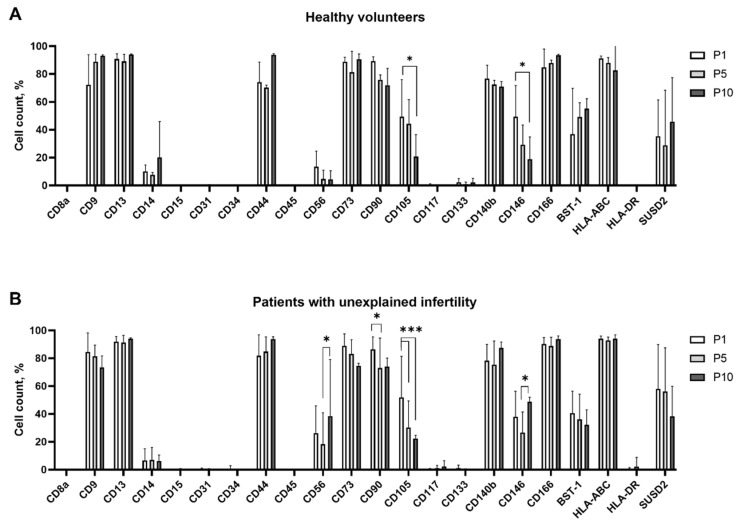
Analysis of cell surface markers of MenSCs. (**A**)—from healthy volunteers, (**B**)—from patients with unexplained infertility. The expression of mesenchymal cell surface markers CD9, CD13, CD44, CD56, CD73, CD90 CD105, CD140b, CD166, BST-1 and SUSD2; hematopoietic and endothelial cell markers CD8a, CD14, CD15, CD31, CD34, CD45, CD117 CD133 and CD146; as well as MHC class I and II cell surface receptors, HLA-DR and HLA-ABC were measured using flow cytometry at different passages (p1, p5 and p10) of MenSCs. Results are presented as mean ± S.D. (n ≥ 5). Note: * denotes a significant difference between MenSCs at p1 and subsequent passages with *p* < 0.05, *** denotes a significant difference with *p* < 0.005, as evaluated using mixed-effects analysis with Tukey’s multiple comparison test.

MenSCs were further characterized by flow cytometry for cell surface marker expression at passages 1, 5 and 10 (Figure 1A,B).

Both studied groups of MenSCs were highly positive for mesenchymal stem cell markers CD9 (tetraspin; 72–85% at p1), CD13 (multifunctional cell surface peptidase; up to 92% at p1), CD44 (homing cell adhesion molecule; up to 94% at p10), CD73 (5′-nucleotidase; up to 89% at p1), CD90 (thymocyte differentiation antigen 1; up to 89% at p1), CD140b (PDGFRβ; up to 78% at p1) and CD166 (activated leukocyte cell adhesion molecule; up to 94% at p10). It is worth mentioning that MenSCs from both studied groups were also positive for bone marrow stromal cell antigen 1 (BST-1), human MHC class I cell surface receptors HLA-ABC and membrane protein SUSD2 (Sushi domain containing 2). No or only negligible expression of hematopoietic and endothelial cell markers (CD14, CD8a, CD15, CD31, CD34, CD45, CD117 and CD133) was detected in MenSCs from healthy volunteers and patients with unexplained infertility.

We observed differences in cell surface marker expression between groups: samples from the “Patients with unexplained infertility” group were characterized by higher expression of CD56 (at p10 vs. p5 and p10 of “Healthy volunteers” group; results significant with *p* < 0.05) and SUSD2 (at p1 vs. p1 and p5 of “Healthy volunteers” group; *p* < 0.05). In addition, changes within groups in CD expression upon cell passaging were also registered. For example, the percentage of cells positive for CD105 diminished during passaging (by 58% from p1 to p10 in “Healthy volunteers” group and by 43% in ”Patients with unexplained infertility” group).

### 2.2. MenSCs Ability to Differentiate into Multilineage Directions

**Figure 2:**
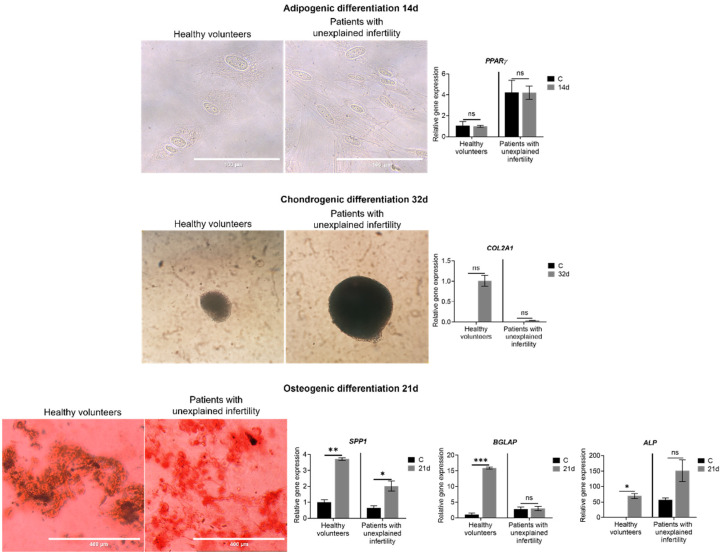
Morphology and expression of genes of MenSCs differentiated in adipogenic, chondrogenic and osteogenic directions. 14d—cells after 14 days of culture in differentiation medium, 32d—cells after 32 days of culture in differentiation medium, 21d—cells after 21 days of culture in differentiation medium. Cells were stained with oil red for assessment of adipogenic differentiation and alizarin red for staining for osteogenic differentiation. Scale bars: 100 µm (40× objective) and 400 µm (10× objective). Gene expression results are presented as mean ± standard deviation. * *p* ≤ 0.05; ** *p* ≤ 0.01; *** *p* ≤ 0.001; ns—not statistically significant, based on *t*-test.

In this study, we aimed to examine cell differentiation in three directions: adipogenic, osteogenic and chondrogenic (Figure 2). Examining the morphology and gene expression of MenSCs during differentiation, we found that the cells of healthy volunteers and patients with unexplained infertility are able to differentiate into multilineage directions.

Specific morphological changes were observed during differentiation to chondrogenic direction—the cells of both groups (healthy vs. infertile) formed three-dimensional spheres after 32 days. During chondrogenic differentiation at day 32, the expression of the *COL2A1* gene, which is characteristic of cartilage formation, was increased very slightly in MenSCs of the healthy volunteers’ group.

We also found that healthy volunteers and patients with pathology MenSCs are able to differentiate in the osteogenic direction: morphological changes and an increase in the expression of genes important for bone formation *ALP, SPP1, BGLAP* was observed after 21 days of culture in differentiation medium (Figure 2).

In the study of adipogenic differentiation after 14 days of culture in the differentiation medium, no changes in morphology of MenSCs were observed, and lipid droplets did not form in the cells of either healthy volunteers or patients with pathology. When studying the expression of the *PPARγ* gene marker of this differentiation, no significant changes were also observed during differentiation. Thus, it can be stated that MenSCs of the studied volunteers and patients are able to successfully differentiate in osteogenic and chondrogenic directions but have low potential for adipocytic differentiation (Figure 2).

### 2.3. Decidualization of MenSCs—Morphology and Gene Expression Changes

**Figure 3:**
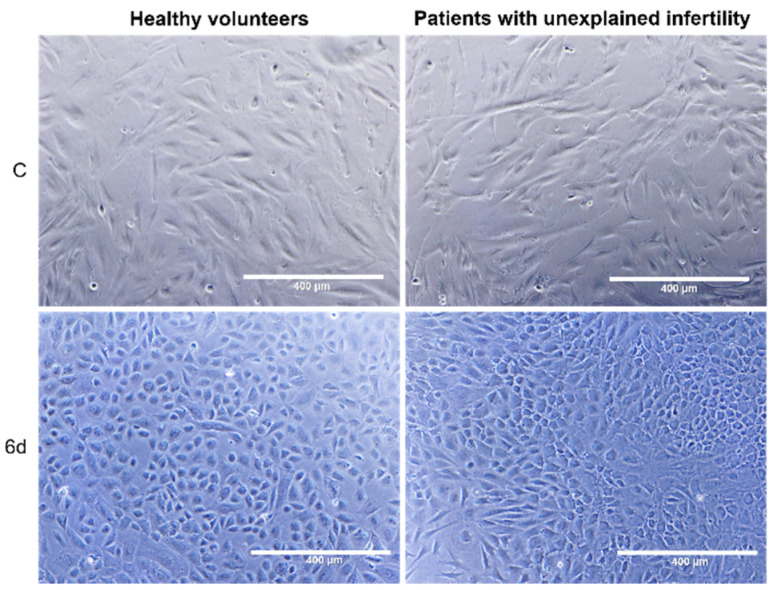
Morphology of menstrual stem cells differentiation into epithelial cells. C—control cells, 6d—cells after 6 days of culture in differentiation medium. Scale bar: 400 µm (10× objective).

MenSCs isolated from healthy volunteers or infertility patients were induced to decidualization by using a combination of 8-brom-cAMP and MPA. After 6 days of treatment, it was found that the MenSCs of both healthy volunteers and patients with pathology were able to differentiate into epithelial cells, specific morphological changes were observed, and the shape of cells changed from spindle-shaped to oval (Figure 3).

In order to fully characterize the MenSCs typical for healthy volunteers and patients with unexplained infertility, the gene expression analysis was performed. Sixteen genes related to decidualization, stemness and angiogenesis, and 11 proteins related to the decidualization process were selected and studied.

**Figure 4:**
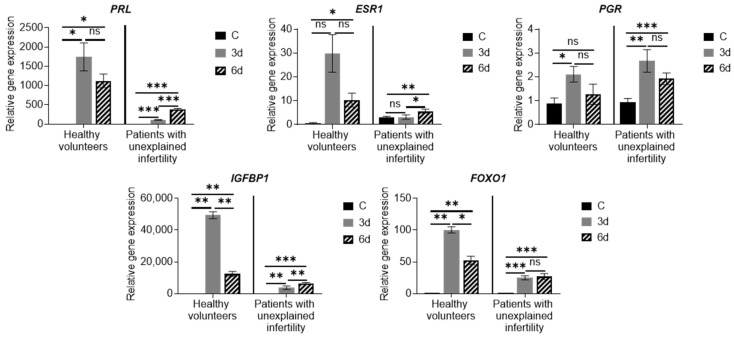
Expression of genes inherent in decidualization. Results are presented as mean ± standard deviation. In the group of healthy volunteers *n* = 2, in patients with unexplained infertility *n* = 4. C—control cells, 3d—cells after 3 days of culture in decidualization medium, 6d—cells after 6 days in differentiation medium. * *p* ≤ 0.05; ** *p* ≤ 0.01; *** *p* ≤ 0.001, ns—not statistically significant, based on *t*-test (Holm–Sidak method).

During the analysis of the expression of the major genetic markers of decidualization, the hormone prolactin (*PRL*), the progesterone receptor gene (*PCR*), the estrogen receptor (*ESR*), insulin-like growth factor binding protein 1 (*IGFBP)* and forkhead box protein O1 (*FOXO1)*, we revealed changes in *PRL, PCR, ESR, IGFBP, FOXO1* expression in time manner and difference in the groups (healthy vs. infertile). We detected downregulation of *PRL, ESR* in patients with unexplained infertility MenSCs compared with healthy volunteers (Figure 4). We determined that the expression of *IGFBP-1* and *FOXO1* was upregulated at the 3-day point of decidualization with further down regulation at the 6-day point in the heathy volunteers’ group. In contrast, *IGFBP-1* expression in infertility patients’ group was very low during all 6 days of decidualization. Thus, the increase in the expression of the main markers of decidualization confirms the results of morphological observation of cell culture in the differentiation medium and shows that MenSCs can differentiate into epithelial cells.

**Figure 5:**
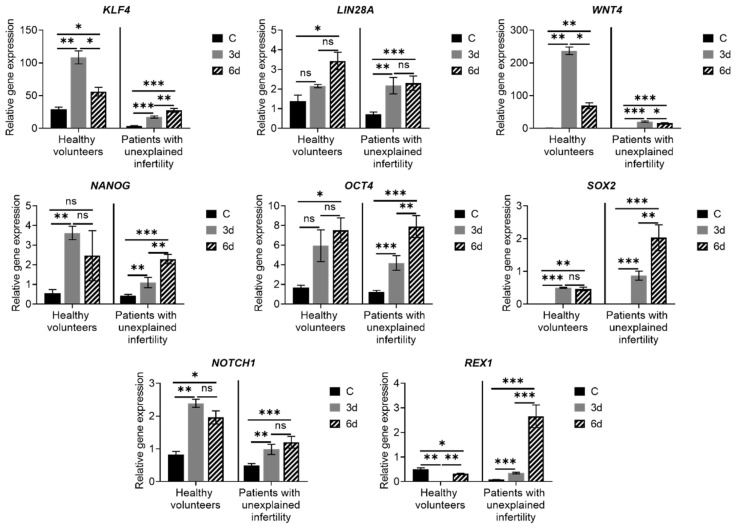
Expression of stemness genes inherent in decidualization. Results are presented as mean ± standard deviation. In the group of healthy volunteers *n* = 2, in patients with unexplained infertility *n* = 4. C—control cells, 3d—cells after 3 days of culture in decidualization medium, 6d—cells after 6 days in differentiation medium. * *p* ≤ 0.05; ** *p* ≤ 0.01; *** *p* ≤ 0.001, ns—not statistically significant, based on *t*-test (Holm–Sidak method).

The expression of genes *SOX2, REX1*, *NOTCH1, NANOG, OCT4, WNT4, KLF4* and *LIN28A* that are essential for maintaining cell pluripotency in healthy volunteers and patients with unexplained infertility MenSCs was analyzed. We determined that the expression of *NOTCH1, NANOG, WNT4, KLF4, OCT4, SOX2* and *LIN28A* was upregulated in differentiated MenSCs cells compared to control cells. Additionally, we found that *WNT4, KLF4* expression level in the healthy volunteers’ group was higher than in the infertility group. Meanwhile, the expression of *REX1* in healthy volunteers’ MenSCs was downregulated and in patients with pathology, it was upregulated (Figure 5). Thus, the increase in the expression of stemness genes important for decidualization indicates that MenSCs of both healthy volunteers and patients with pathology has the necessary expression of genes to support decidualization.

**Figure 6:**
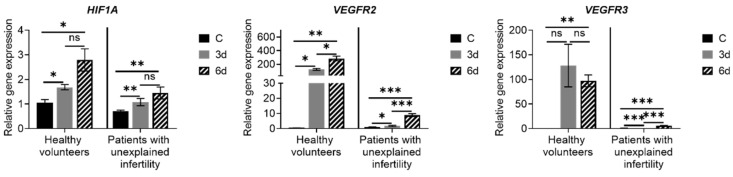
Expression of angiogenesis-related genes. Results are presented as mean ± standard deviation. In the group of healthy volunteers *n* = 2, in patients with unexplained infertility *n* = 4. C—control cells, 3d—cells after 3 days of culture in decidualization medium, 6d—cells after 6 days in differentiation medium. * *p* ≤ 0.05; ** *p* ≤ 0.01; *** *p* ≤ 0.001, ns—not statistically significant, based on *t*-test (Holm–Sidak method).

Studies of the expression of angiogenesis genes *HIF1A, VEGFR-2* and *VEGFR-3,* which are involved in the regulation of angiogenesis, have shown that in MenSCs of healthy volunteers and patients with pathology expression of these genes has increased during decidualization (Figure 6). However, we detected differences in this expression in the healthy vs. infertile group: *HIF1A, VEGFR-2* and *VEGFR-3* expression was at the higher level in the healthy volunteers group. The obtained results suggest that during the decidualization, MenSCs could participate in the process of blood circulation between the fetus and the mother and in the process of ensuring activity.

### 2.4. Changes in Protein Levels of MenSCs during Decidualization

**Figure 7:**
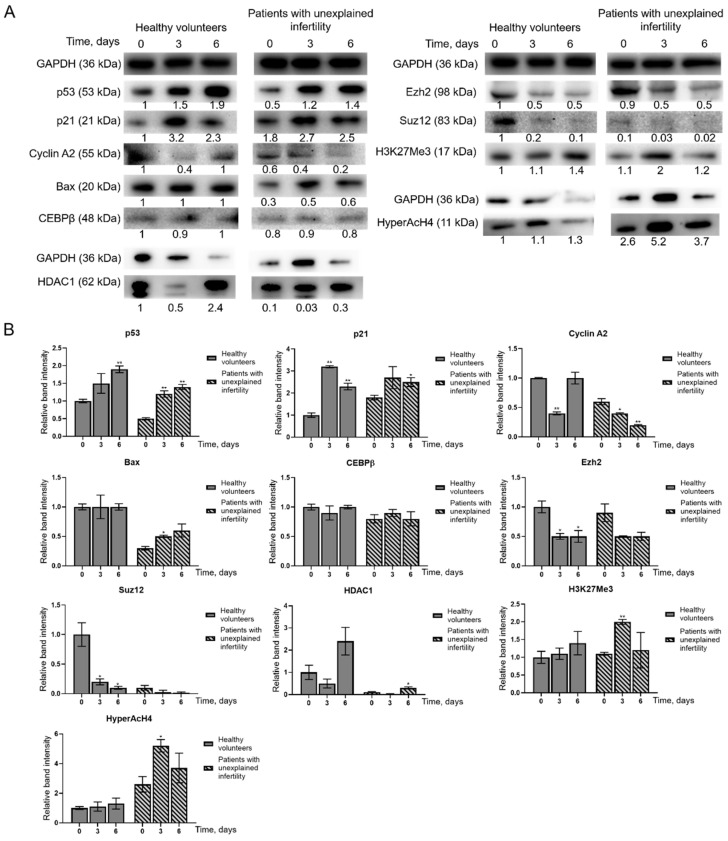
Evaluation of changes in protein levels. In each study group *n* = 2. (**A**) C—control cells, 3—proteins isolated from MenSCs after 3 days in differentiation medium, 6—proteins isolated from MenSCs after 6 days in differentiation medium. GAPDH is a control protein. The intensity of the protein bands was estimated by the ImageJ program, and the levels of each protein were calculated according to the GAPDH. (**B**) Densitometric analysis graphs of relative band intensity of detected protein levels as measured using ImageJ software and normalized to the GAPDH loading control; results are mean ± S.D. * *p* ≤ 0.05; ** *p* ≤ 0.01, based on *t*-test.

In this study, proteins involved in cell cycle regulation and apoptosis (p53, p21, Cyclin A2, Bax, CEPBβ) and epigenetic regulation (Ezh2, Suz12, HDAC1, H3K27me3, HyperAcH4) were analyzed by Western blot (Figure 7A). Densitometric analysis graphs of relative band intensity of detected protein levels was measured and presented in Figure 7B. The levels of p53 and p21 proteins in the cells of healthy volunteers and infertile patients were found to increase during decidualization in comparison to untreated cells. Bax level in healthy volunteers’ cells remain constant, while Cyclin A2 temporally decreased after 3 days of decidualization induction recovering to control level after 6 days of differentiation. In MenSCs of patients with unexplained infertility, the levels of these proteins change during decidualization, with Bax levels increased and Cyclin A2 decreased. CEBPβ levels in MenSCs of healthy volunteers and patients with unexplained infertility remain more or less constant for 6 days of decidualization. The levels of epigenetic regulatory proteins Ezh2, Suz12 and HDAC1 changed differently: Ezh2 and Suz12 decreased and HDAC1 decreased only after 3 days of differentiation. The levels of modified histones H3K27me3 and HyperAcH4 slightly increased during differentiation both in the cells of healthy volunteers and patients with unexplained infertility.

### 2.5. Changes in MenSCs’ Secreted Proteins during Decidualization

Using mass spectrometry technique, we analyzed the proteins that were secreted by the MenSCs of healthy volunteers and patients with unexplained infertility. A total of 187 human cell-specific MenSCs’ secreted proteins were detected in samples (Table S4).

**Figure 8:**
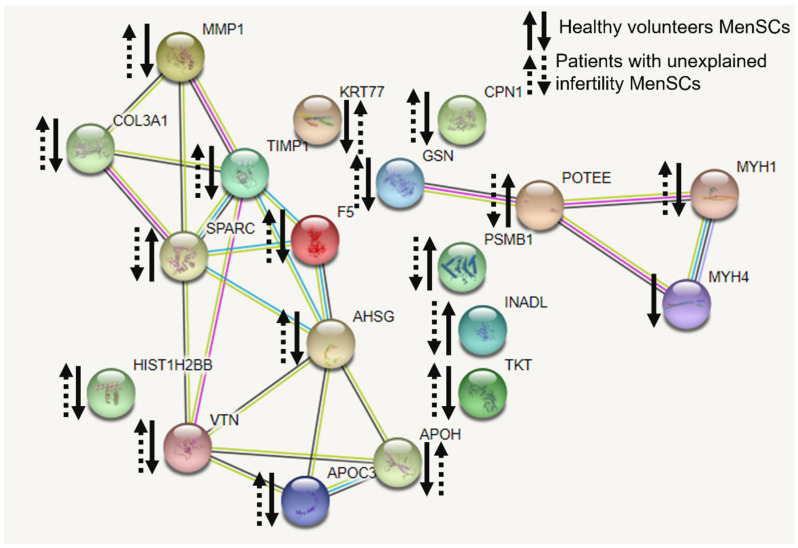
Interaction networks of secreted proteins from menstrual stem cells of healthy volunteers and patients with unexplained infertility during decidualization. The ↑↓ symbols—upregulation or downregulation of proteins in the samples. The interaction networks of secreted proteins and their involvement in different biological processes were identified using the STRING database (https://string-db.org/cgi/network?taskId=bQ56qZB0Kkow&sessionId=bcvMfLgxWFKc, retrieved 20 January 2021).

We analyzed the interactions between the identified MenSCs’ secreted proteins and determined their involvement in various cellular processes. We grouped proteins according to their involvement in decidualization or other processes important for decidualization success, such as actin cytoskeletal regulation, estrogen and relaxin signaling pathways and cell–cell interactions (Table S5). The change in protein levels was calculated by comparing the amount secreted by control MenSCs and MenSCs decidualized for 6 days to find out how many times the protein levels increased or decreased during decidualization. Of the 187 proteins, 19 proteins of MenSCs were differently secreted during decidualization in healthy volunteers and patients with unexplained infertility samples (Figure 8).

Functions of differentially secreted proteins from menstrual cells in healthy volunteers and infertile patients during decidualization were defined, and the interaction networks of secreted proteins and their involvement in different biological processes were identified using the STRING database. Differently secreted proteins regarding the secretome of volunteers and were found to be involved in cholesterol metabolism, PPAR signaling pathway, complement cascade, tight junction between cells and regulation of the relaxin signaling pathway, etc. (Table S6).

## 3. Discussion

Mesenchymal stem cells hypothetically can be obtained from almost any tissue within the human body, for example bone marrow, adipose tissue, dental pulp, exfoliated deciduous teeth, perinatal derivatives such as amniotic fluid and placenta, peripheral blood, synovium and synovial fluid, endometrium, skin, muscle, etc. Still, there are practical limitations concerning the difficulty and invasiveness of the procurement process and various donor characteristics [12,13,14,15]. To study the properties of MenSCs, we analyzed the growth rate, cell surface marker expression and proteome profile of MenSCs characteristic for healthy volunteers and patients with unexplained infertility. Additionally, the potential of MenSCs for decidualization and changes in gene expression, intracellular and secreted proteins during decidualization were revealed.

In 2007, Meng and coauthors identified a new source of stem cells, menstrual blood, and named the stem cells found in it endometrial regenerative cells, now called menstrual stem cells. Despite the similar phenotypes and characteristics of EnSCs and MenSCs, the therapeutic effects and mechanisms of these cells are unique [11]. In this study, we found that the cell culture doubling time is about 3.2–3.4 days. Analysis of the proliferation of MenSCs in healthy volunteers and patients with unexplained infertility suggests that the rate of proliferation is not related to pathology but is determined by the individual biological characteristics of each patient.

The results of our study demonstrated that MenSCs from both groups, healthy volunteers, as well as patients with unexplained infertility, could be characterized by the high expression of mesenchymal stem cell markers (CD9, CD44, CD73, CD90 and CD166). Our data also coincide with the work of Sheikholeslami et al. (2021) who showed that MenSCs from healthy and infertile women (having endometriosis or polycystic ovary syndrome) do possess similar CD marker patterns [16]. However, our study revealed that MenSCs obtained from patients with unexplained infertility are different from MenSCs from healthy volunteers in more pronounced expression of mesenchymal cell surface markers CD56, SUSD2. These observations illustrate subtle, though significant, variations in characteristics of MenSCs, obtained from different sources.

Decidualization, or differentiation in the direction of the epithelium, is the restructuring of endometrial tissue necessary to create favorable conditions for the implantation of a fertilized oocyte. In order to use MenSCs for the treatment of infertility associated with endometrial disorders, it is necessary to elucidate the potential of decidualization of these cells [17]. Elevated progesterone and cellular cAMP levels after ovulation are known to activate the transcription factor Foxo1 in EnSCs, which arrests the cell cycle, and cells differentiate into decidualized cells that control embryo implantation [18]. In this study, we showed that in vitro treatment of MenSCs with medium enriched with 8-bromo-cAMP and MPA successfully induced the decidualization process. We also studied the changes in MenSCs that occur during decidualization.

Various scientific groups investigated and identified molecular mechanisms for regulating endometrial decidualization in vitro using cultured EnSCs with functional progesterone and estrogen receptors. Morphological signs of differentiation and expression of decidualization markers, such as prolactin (*PRL*) and insulin-like growth factor binding protein 1 (*IGFBP-1*), have been observed after 12 days of progesterone treatment of EnSCs [19,20,21]. An increase in cyclic adenosine monophosphate (cAMP) levels in cells was also found to occur. Ovarian hormones as well as relaxin, a corticotropin-releasing factor, and prostaglandin E2 promote the accumulation of cellular cAMP. In EnSCs cultures, exposure to cAMP alone increases *PRL* expression, which is further enhanced if cells are exposed to cAMP and progesterone. A critical pathway for the regulation of decidualization consists of progesterone and proteins regulated by progesterone and/or cAMP, such as HOXA10, FOXO1, signal transducers and transcriptional activators, and Hand2, which plays an important role in endometrial receptivity [22]. Thus, the increase in the expression of the main markers of decidualization confirms the results of morphological observation of cell culture in the differentiation medium and shows that MenSCs can differentiate into epithelial cells.

Examining the expression of MenSC genes during decidualization, we found that the expression of the main decidualization markers *PRL, PCR, ESR, IGFBP* and *FOXO1* is upregulated, the expression of *NOTCH1, NANOG, WNT4, KLF4, OCT4, SOX2* and *LIN28A* increased, and expression of angiogenesis-related genes HIF1A, VEGFR-2 and VEGFR-3 is upregulated as well. Many genes and proteins are responsible for endometrial development and regeneration, and the best known are Wnt, c-kit (CD117), Oct-4, CD34/Klf4 and Musashi-1. The WNT gene activates a signaling cascade, binds to cell surface markers in the Frizzled family and determines cell fate.

In a study of changes in protein levels during MenSCs decidualization, we found that p53 and p21 protein levels increased in MenSCs. Bax and Cyclin A2 levels in MenSCs from healthy volunteers remain constant, HDAC1 levels increase, and in MenSCs from patients with infertility change: Bax levels decrease and Cyclin A2 levels increase. The levels of epigenetic regulatory proteins Ezh2, Suz12 and HDAC1 decreased during MenSCs decidualization, while the levels of modified histones H3K27me3 and HyperAcH4 increased. Grimaldi and colleagues in 2011 showed that the amount of histone methyltransferase enhancer Ezh2 decreases during decidualization, resulting in a decrease in the level of H3K27me3 in the proximal promoters of the major decidualization marker genes *PRL* and *IGFBP1*. Demethylation of H3K27me3 has been associated with acetylation of the same lysine groups, indicating active conversion from transcriptionally inactive to transcriptionally active chromatin [23]. In 2012, Estella and the group analyzed the effect of histone acetylation on the expression of tissue remodeling enzymes and EnSC activity related to the control of trophoblast invasion. Treatment of EnSCs with the HDAC inhibitor trichostatin A (TSA) increased the expression of Timp-1 and Timp-3 and decreased the expression of metalloproteinases. Acetylation of histones has been shown to disturb the balance of modulators of the extracellular filler and to limit the invasion of trophoblasts [24]. Sakai and colleagues in 2003 showed that the HDAC inhibitor TSA increases the levels of the decidualization markers IGFBP-1 and prolactin in a dose-dependent manner via 17β-estradiol (E2) and progesterone (P4). Morphological changes similar to decidual transformation also manifest more strongly in the decidualization medium with the addition of TSA [25]. In a 1996 study of apoptosis in rat decidualized tissues, Akcali and colleagues found that control of progesterone and estrogen for endometrial differentiation and possible apoptosis includes control of *BCL-2* gene family expression. Exposure of rat ovaries to 3.5 mg medroxyprogesterone acetate and 200 ng estradiol decreased *BCL-2* expression. In situ analysis revealed a cell type-specific increase in Bax expression after hormonal treatment and decidualization. Data suggest that the balance between Bax and Bcl-2 expression is altered during stromal cell differentiation. Increased expression of Bax is characteristic of apoptosis, and apoptosis plays a significant role in placental development [26].

It has been found by various groups of researchers that even before the stage of direct embryo attachment to the uterus, there is a precisely regulated exchange of cellular signaling molecules between the embryo and the maternal endometrium. Decidualized EnSCs secrete many different factors, including immunologically active ones, that ensure this maternal connection. However, the cell secretion profile is quite complex and depends strictly on which stage of the menstrual cycle the cells are being examined. EnSC transition into the G0/G1 phase begins with increased secretion of various inflammatory chemokines, cytokines, C-reactive protein and other inflammatory mediators (e.g., IL-2, IL-12 and Interferon-γ). These pro-inflammatory factors are thought to promote endometrial receptivity and early stages of implantation by auto/paracrine activity, as they increase the expression of major genes required for embryo implantation, including growth factors (e.g., HB-EGF), cytokines (e.g., LIF, IL1β, PROKL and IL-11) and various morphogens (e.g., IHH, WNT4, BMP2). The phase of such pro-inflammatory secretion is tightly controlled and usually lasts for 2 to 4 days. The time-limiting factor in this case is a change in secretion profile. It is the cells that begin to secrete anti-inflammatory soluble factors and receptors and the potent anti-inflammatory hormone cortisol. Such a transition from the anti-inflammatory stage of the process is characterized by inhibition of many anti-inflammatory genes below basal levels and induction of various anti-inflammatory cytokines, including IL-4, IL-5 and IL-10 [27]. In our study, detected secreted proteins were identified to be involved in the regulation of the actin cytoskeleton, the regulation of estrogen and relaxin signaling pathways, the provision of cell–cell connections and the management of inflammatory processes. Based on the literature, these identified secretory proteins confirm the successful decidualization of MenSCs. Several other study groups analyzed secreted proteins during pregnancy. It is known that an appropriate transcriptional profile in the placenta and perinatal membranes is required for successful implantation and pregnancy [28]. Our study showed that secretion of proteins COL3A1, TIMP1 and MMP1, and PPAR taking part in relaxin signaling pathway decreased in healthy volunteers’ MenSCs while increased in patients with unexplained infertility. Matrix metalloproteinase (MMP)-mediated extracellular matrix degradation plays a key role in normal growth and remodeling of fetal membranes throughout gestation and are involved in the weakening and subsequent rupture of the fetal membranes at the time of labor [28]. Sundrani et al., 2012, discovered that placental MMP-1 levels are higher in women delivering preterm as compared to those delivering at term [29]. So far, the regulation mechanisms involved in matrix metalloproteinase expression and the motility of human endometrial and decidual stromal cells during decidualization remain unclear [30]. Nevertheless, the estrogen signaling pathway in which the matrix metalloproteinase is involved should be considered for the treatment of infertility of unknown origin. For the decidualization and successful implantation, relaxin action with an accompanied increase in estrogen-associated factors is important [31]. Accordingly, the main identified proteins demonstrate that at least relaxin and estrogen signaling pathways and the proteins involved in them are important for successful conception. Data suggest that these identified secretory proteins could be one of underlying causes for unexplained infertility.

## 4. Materials and Methods

### 4.1. Patient Recruitment

We conducted an interventional prospective cohort study. Patients were recruited from 2017 to 2020 at the Vilnius University Hospital Santaros Klinikos Obstetrics and Gynecology Center Santaros Fertility Center. Females with unexplained infertility or poor ovarian reserve were enrolled in this study. Protocols approved by the Ethics Committee of Biomedical Research of Vilnius District, No 158200-18/7-1049-550. The inclusion criteria were as follows: (a) age of woman at the time of enrollment is 30–35 years (average—35 years old), (b) the average duration of infertility—4.2 years (range 1.5–6.5 years), (c) couples with unexplained infertility diagnosis are confirmed after laboratory and instrumental investigation, (d) woman confirms the participation in the study. The exclusion criteria were as follows: (a) woman is pregnant or breastfeeding, (b) oncological disease was confirmed for woman during the last three years, (c) other infertility causes, except unexplained infertility, are confirmed, (d) woman is addicted to alcohol or other substances, (e) pregnancy is contraindicated, (f) uncontrolled endocrine or other medical conditions, such as prolactinemia or thyroid diseases.

Healthy volunteers were enrolled in the study in age of 18–45 years at the time of enrollment. The female volunteers had regular menstrual cycle (range 24–35) with normal hormonal status and no history of any other surgeries, diseases, smoking, medications, alcohol and other substance abuse.

### 4.2. Menstrual Blood Sample Collection and MenSCs Isolation and Cultivation

Menstrual blood samples were collected with a DivaCup during the second and third days of menstruation. Once received in the laboratory, menstrual blood samples were transferred into a 50 mL tube and treated with 50 U/mL collagenase II (Sigma-Aldrich, St. Louis, MO, USA) for 20 min at 37 °C in a humidified 5% CO_2_. MenSC isolation was immediately performed using the “Ficoll-Paque™ premium” (Sigma-Aldrich, St. Louis, MO, USA) according to manufacturer’s protocol. After isolation MenSC washed in phosphate-buffered saline (PBS) (Gibco, Thermo Fisher Scientific, Waltham, MA, USA) twice and suspended in growth medium DMEM/F12 medium (Gibco, Thermo Fisher Scientific, Waltham, MA, USA) supplemented with 10% FBS (Gibco, Thermo Fisher Scientific, Waltham, MA, USA) and 1% penicillin (100 U/mL)—streptomycin (100 µg/mL) solution (Gibco, Thermo Fisher Scientific, Waltham, MA, USA) and, then, seeded into a 75 cm^2^ plastic cell culture flasks and cultivated at 37 °C in a humidified 5% CO_2_ atmosphere. After 3–5 days of culture, non-adherent cells were washed away with PBS (Gibco, Thermo Fisher Scientific, Waltham, MA, USA) leaving behind adherent fibroblastic cells growing in clusters. The growth medium was replaced every 3 days.

### 4.3. Proliferation, Doubling Time and Viability Assay

MenSCs proliferation and viability were evaluated by trypan blue exclusion test. Cells were mixed with 0.2% of trypan blue dye (final concentration) (Pharmacia LKB, Uppsala, Sweden). Viable and dead (blue colored) cell numbers were determined by counting the cells in a hemocytometer under the light microscope. Cell doubling time was estimated by monitoring cell proliferation and calculated using the online tool https://doubling-time.com, retrieved November 15, 2020. Doubling time is expressed in days and shows how long it takes to increase the number of cells by 2 times.

### 4.4. Flow Cytometry Analysis

For phenotypical characterization of MenSCs, 0.05 × 10^6^ cells for one assay were collected by centrifugation at 500× *g* for 5 min. Pelleted cells were washed twice in PBS supplemented with 1% bovine serum albumin (BSA) (Sigma-Aldrich, St. Louis, MO, USA). Then, cells were suspended in 50 μL PBS with 1% BSA and incubated with the antibodies (dilution ratio 1:25) against cell surface markers in the dark at 4 °C for 30 min. Antibodies are presented in Table S1. After incubation samples were washed twice with PBS with 1% BSA. Finally, cells were suspended and fixated in 200 μL PBS with 1% paraformaldehyde (PFA) (Sigma-Aldrich, St. Louis, MO, USA) and analyzed using Partec flow cytometer (Sysmex Corporation, Cobe, Hioko, Japan) with Flowing Software 2 software.

### 4.5. Adipogenic, Chondrogenic and Osteogenic Differentiation

MenSCs were cultured at 80–90% confluence and subsequently differentiated with differentiation medium at 37 °C in 5% CO_2_. Briefly, for differentiation assays, after cultivation, the growth medium was changed to either adipogenic differentiation medium: 4.5 g/L glucose DMEM (Gibco, Thermo Fisher Scientific, Waltham, MA, USA) +1% penicillin–streptomycin (Gibco, Thermo Fisher Scientific, Waltham, MA, USA) +1 μM dexamethasone (Cayman Chemical Company, Ann Arbor, MI, USA) +60 µM indomethacin (Sigma-Aldrich, St. Louis, MO, USA) +0.5 mM IBMX (Sigma-Aldrich, St. Louis, MO, USA), for 14 days; osteogenic differentiation medium: 1 g/L glucose DMEM (Gibco, Thermo Fisher Scientific, Waltham, MA, USA) +1% penicillin– streptomycin (Gibco, Thermo Fisher Scientific, Waltham, MA, USA) +10% FBS (Gibco, Thermo Fisher Scientific, Waltham, MA, USA) +0.1 μM dexamethasone (Cayman Chemical Company, Ann Arbor, MI, USA) +50 μg/mL ascorbic acid (Cayman Chemical Company, Ann Arbor, MI, USA) +10 mM β-glycerophosphate (Cayman Chemical Company, Ann Arbor, MI, USA), for 21 days; chondrogenic differentiation medium: 4.5 g/L glucose DMEM (Gibco, Thermo Fisher Scientific, Waltham, MA, USA) +1% penicillin–streptomycin (Gibco, Thermo Fisher Scientific, Waltham, MA, USA) +0.1 μM dexamethasone (Cayman Chemical Company, Ann Arbor, MI, USA) +50 μg/mL ascorbic acid (Cayman Chemical Company, Ann Arbor, MI, USA) +1X ITS (PeproTech, London, UK) +1 mM sodium pyruvate (Genaxxon bioscience, Ulm, Germany) +0.35 mM proline (Genaxxon bioscience, Ulm, Germany), for 32 days, the induction medium was replaced every 3 days. Control cells were cultured in growth medium. At the end of the induction periods, the cells were washed and fixed. For cell staining, MenSCs were seeded into a 4-well (3.85 cm^2^) plate (Nunc, Thermo Fisher Scientific, Roskilde, Denmark) at a 2 × 10^4^ cells/cm^2^ density. Adipogenic differentiation was confirmed by Oil Red O (Sigma-Aldrich, St. Louis, MO, USA) staining and osteogenic differentiation was confirmed by Alizarin Red (Sigma-Aldrich, St. Louis, MO, USA) staining and differentiation-specific genetic marker expression.

### 4.6. Decidualization Induction

MenSCs at 1 × 10^4^ cells/cm^2^ density were cultured to 80–90% confluence and differentiated with decidualization induction medium: phenol red free RPMI 1640 (Hyclone Laboratories, Logan, UT, USA) +2% charcoal stripped FBS (Gibco, Thermo Fisher Scientific, Waltham, MA, USA) +1% penicillin–streptomycin (Gibco, Thermo Fisher Scientific, Waltham, MA, USA) +0.5 mM 8-brom-cAMP (Cayman Chemical Company, Ann Arbor, MI, USA) +1 µM medroxyprogesterone acetate (MPA) (Cayman Chemical Company, Ann Arbor, MI, USA) at 37 °C in 5% CO_2_ for 6 days. Differentiation was confirmed by observation of cell morphology changes with light microscope “EVOS™” (Thermo Fisher Scientific, Waltham, MA, USA).

### 4.7. Gene Expression Analysis by RT-qPCR

Total RNA was purified using TRIzol reagent (Invitrogen, Carlsbad, CA, USA), cDNA was synthesized using SensiFAST™ cDNA Synthesis Kit (Bioline, Memphis, TN, USA), and qPCR was performed using SensiFAST™ SYBR^®^ No-ROX Kit (Bioline Memphis, TN, USA) on the RotorGene 6000 system (Corbett Life Science, QIAGEN, Hilden, Germany). Primer sequences (Metabion international AG, Planegg/Steinkirchen, Germany) are presented in Table S2. mRNA levels were normalized to *GAPDH* expression. The reference gene was selected using the NormFinder tool (Aarhus University Hospital, Aarhus, Denmark). Three candidate genes were tested with stability values of 0.143 for *GAPDH*, 0.411 for *HPRT1* and 1.224 for *TUBB*. Relative gene expression was calculated using the ΔΔCt method.

### 4.8. Protein Isolation and Immunoanalysis

MenSCs (1–2 × 10^6^) were harvested, washed twice in ice-cold PBS, and 0.1 volume of benzonase (Merck, Darmstadt, Germany) was added to the tubes with patient cells and incubated for 30 min on ice, then 1 volume of 2× SDS lysis buffer (125 mM Tris, pH 6.8; 4% SDS, 600 mM DTT, 5% glycerol) and up to 20 volumes of 1× SDS lysis buffer were added. The mixture in the tubes was fragmented with an insulin syringe until non-viscous. After lysis, the tubes were incubated at 95 °C for 5 min. The ready-to-use samples were immediately subjected to electrophoresis or stored in a freezer at −20 °C.

Proteins were fractionated on a 7–15% polyacrylamide gradient SDS/PAGE gel using Tris-glycine buffer. After protein transfer to a PVDF membrane (Immobilon P; Millipore, Billerica, MA, USA), the membrane was blocked with “AdvanBlock-Chemi Antibody-antigen enhancing and blocking solution” (Advansta, San Jose, CA, USA), washed in PBS–Tween-20 and probed with the primary antibody (Table S3) in accordance with the manufacturer’s instructions. The membrane was subsequently washed four times with PBS–Tween-20 and then incubated with horseradish peroxidase (HRP)-linked secondary antibody for 1 h at room temperature. GAPDH was used as loading control. “Clarity Western ECL Substrate” (BIORAD, Hercules, CA, USA) was used for chemiluminescent detection. Signal detection was carried out on ChemiDoc XRS+ System (BIORAD, Hercules, CA, USA). Quantitative evaluation was performed using ImageJ software.

### 4.9. Secretome Analysis by Mass Spectrometry

MenSCs’ culture medium is aspirated and collected in 5 mL tubes and centrifuged for 10 min at 2000× *g* to remove cells and cell debris. After centrifugation, the supernatant is collected and filtered through a 0.22 μm filter. After filtration, the samples are cleaned and concentrated using ProteoMiner^TM^ Sequential Elution Large-Capacity kit (Bio-Rad Laboratories, Hercules, CA, USA) according to the manufacturer’s instructions. The secretome protein concentration was measured using the Pierce Detergent Compatible Bradford Assay Kit (Thermo Fisher Scientific, Waltham, MA, USA) according to the manufacturer’s instructions. The secretome proteins were trypsinized according FASP protocol, as described by Wisniewski et al. [32]. Briefly, proteins were diluted in 8 M urea; following two washes with urea, proteins were alkylated with 50 mM iodoacetamide (GE Healthcare Life Sciences, MA, USA). Protein concentrators were washed twice with urea and twice with 50 mM NH_4_HCO_3_. Proteins were digested overnight with TPCK Trypsin 20233 (Thermo Scientific, Vilnius, Lithuania). After overnight digestion, peptides were collected from the concentrators by centrifugation at 14,000× *g* for 10 min and additionally eluted using 20% CH_3_CN. The eluates were combined, acidified with 10% CF_3_COOH and lyophilized in vacuum centrifuge. The lyophilized peptides were redissolved in 0.1% formic acid.

### 4.10. LC-MS-Based Protein Identification

Liquid chromatographic (LC) analysis was performed in a Waters Acquity ultra performance LC system (Waters Corporation, Wilmslow, UK). Peptide separation was performed on an ACQUITY UPLC HSS T3 250 mm analytical column. Data were acquired using Synapt G2 mass spectrometer (MS) and Masslynx 4.1 software (Waters Corporation) in positive ion mode using data-independent acquisition (*UDMS*^E^). Raw data were lock mass-corrected using the doubly charged ion of [Glu1]-fibrinopeptide B (*m*/*z* 785.8426; [M+2H]2+). Raw data files were processed and searched using ProteinLynx Global SERVER (PLGS) version 3.0.1 (Waters Corporation, UK). Data were analyzed using trypsin as the cleavage protease; one missed cleavage was allowed, and fixed modification was set to carbamidomethylation of cysteines; variable modification was set to oxidation of methionine. Minimum identification criteria included 1 fragment ions per peptide, 3 fragment ions and one peptide per protein. The following parameters were used to generate peak lists: (i) low energy threshold was set to 150 counts, (ii) elevated energy threshold was set to 50 counts, (iii) intensity threshold was set to 750 counts. UniprotKB/SwissProt human databases (2020-09-24) were used. Protein quantification was calculated using the ISOQuant software. The obtained results of mass spectrometry analysis were also analyzed with the online tool STRING (String consortium, https://string-db.org, retrieved 20 January 2021).

### 4.11. Statistical Analysis

Data are expressed as mean ± standard deviation (S.D.). T-test (Holm–Sidak method) was used to calculate the significance of difference between samples; significance was set at *p* ≤ 0.05 (*); *p* ≤ 0.01 (**); *p* ≤ 0.001 (***).

## 5. Conclusions

In up to 20 percent of couples who seek medical attention for infertility, the cause of their problem remains unexplained despite extensive hormonal and anatomic evaluation. Extensive analysis of these situations showed that only embryo quality cannot define pregnancy success rate. In addition to embryo quality, it could be hypothesized that women with unexplained infertility diagnosis have abnormalities in endometrial function, including the receptivity of the endometrium. According to the results, we suggest that stem cells of endometrial origin could be an alternative source of cell therapy, including the improvement of endometrium function. In this way, it could facilitate the treatment of reproductive systems diseases, such as infertility.

## Data Availability

The data presented in this study are available on request from the corresponding author.

## Supplementary Materials

The following are available online at https://www.mdpi.com/article/10.3390/ijms22136774/s1.
