# Supplementary material for: Menstrual Blood-Derived Endometrial Stem Cells’ Impact for the Treatment Perspective of Female Infertility
Source: Int J Mol Sci. 2021 Jun 24;22(13):6774. doi: 10.3390/ijms22136774 (PMC8268036; doi:10.3390/ijms22136774)
Supplement: Supplementary file 1 [file ijms-22-06774-s001.zip › Table S1.pdf]

Table S1. Antibodies used for Flow cytometry analysis

Antibody	Fluorescent probe	Isotype control	Manufacturer
**CD31**	488	IgG1 κ	Biolegend, San Diego, California, USA
**HLA-DR**	488	IgG2a κ	Biolegend, San Diego, California, USA
**HLA-ABC**	488	IgG2a κ	Biolegend, San Diego, California, USA
**CD14**	488	IgG1 κ	Biolegend, San Diego, California, USA
**CD4**	APC	Mouse IgG1	Exbio, Vestec, Czech Republic
**CD16**	APC	Mouse IgG1 κ	Exbio, Vestec, Czech Republic
**CD44**	APC	IgG1 κ	Biolegend, San Diego, California, USA
**CD105**	APC	Mouse IgG2a	Exbio, Vestec, Czech Republic
**CD117**	APC	Mouse IgG1 κ	Exbio, Vestec, Czech Republic
**SUSD2**	APC	IgG1 κ	Biolegend, San Diego, California, USA
**BMSC**	APC	IgG1	Biolegend, San Diego, California, USA
**CD8**	APC	Mouse IgG2a	Exbio, Vestec, Czech Republic
**CD90**	APC	Mouse IgG1 κ	Exbio, Vestec, Czech Republic
**CD56**	APC	IgG1 κ	Biolegend, San Diego, California, USA
**CD133**	APC	IgG1 κ	Biolegend, San Diego, California, USA
**CD146**	APC	IgG1 κ	Biolegend, San Diego, California, USA
**CD13**	APC	Mouse IgG1	Exbio, Vestec, Czech Republic
**CD9**	FITC	Mouse IgG1	Exbio, Vestec, Czech Republic
**CD45**	FITC	IgG1 κ	BD Pharmingen, San Jose, California, USA
**CD73**	FITC	Mouse IgG1 κ	Exbio, Vestec, Czech Republic
**CD34**	FITC	IgG2a, κ	Biolegend, San Diego, California, USA
**CD3**	PE	Mouse IgG2b	Exbio, Vestec, Czech Republic
**CD19**	PE	Mouse IgG1	Exbio, Vestec, Czech Republic
**CD15**	PE	Mouse IgM	Exbio, Vestec, Czech Republic
**CD166**	PE	IgG1 κ	Biolegend, San Diego, California, USA
