# Supplementary material for: Menstrual Blood-Derived Endometrial Stem Cells’ Impact for the Treatment Perspective of Female Infertility
Source: Int J Mol Sci. 2021 Jun 24;22(13):6774. doi: 10.3390/ijms22136774 (PMC8268036; doi:10.3390/ijms22136774)
Supplement: Supplementary file 1 [file ijms-22-06774-s001.zip › Table S2.pdf]

**Table S2.** Primers used for RT-qPCR analysis

| Gene name | Forward and reverse primers (5’ –> 3’) | Product (bp) |
|---|---|---|
| *ALPL* | F: AGCCCTTCACTGCCATCCTGT<br>R: ATTCTCTCGTTCACCGCCCAC | 68 |
| *COL2A1* | F: CTGGTGATGATGGTGAAG<br>R: CCTGGATAACCTCTGTGA | 130 |
| *ESR1* | F: TGGGAATGATGAAAGGTGGGATAC<br>R: AGGTTGGCAGCTCTCATGTC | 130 |
| *FOXO1* | F: TCGTCATAATCTGTCCCTACACA<br>R: CGGCTTCGGCTCTTAGCAAA | 168 |
| *GAPDH* | F: AGTCCCTGCCACACTCAG<br>R: TACTTTATTGATGGTACATGACAAGG | 123 |
| *HIF1A* | F: CCAACAGTAACCAACCTCAG<br>R: TCCTGTGGTGACTTGTCCTT | 302 |
| *IGFBP1* | F: TTTTACCTGCCAAACTGCAACA<br>R: CCCATTCCAAGGGTAGACGC | 108 |
| *KLF4* | F: CACATTAATGAGGCAGCCACC<br>R: AAGTCGCTTCATGTGGGAGAG | 169 |
| *LIN28A* | F: TTGTCTTCTACCCTGCCCTCT<br>R: GAACAAGGGATGGAGGGTTTT | 151 |
| *NANOG* | F: AGATGCCTCACACGGAGACT<br>R: GTTTGCCTTTGGGACTGGTG | 96 |
| *NOTCH1* | F: GGTGAGACCTGCCTGAATG<br>R: GTTGGGGTCCTGGCATC | 102 |
| *OCT4* | F: CGAGAAGGATGTGGTCCGAG<br>R: CAGAGGAAAGGACACTGGTC | 136 |
| *BGLAP* | F: CACTCCTCGCCCTATTGGC<br>R: CCCTCCTGCTTGGACACAAAG | 112 |
| *SPP1* | F: GAAGTTTCGCAGACCTGACAT<br>R: GTATGCACCATTCAACTCCTCG | 91 |

| | | |
|---|---|---|
| *PGR* | F: CAGCCAGAGCCCACAATACA<br>R: GTTGTGCTGCCCTTCCATTG | 148 |
| *PPARγ* | F: CGACCAGCTGAATCCAGAGT<br>R: TTGCCAAGTCGCTGTCATCT | 554 |
| *PRL* | F: TGACCCTTCGAGACCTGTTTG<br>R: CTTGCTCCTTGTCTTCGGG | 177 |
| *REX1* | F: GCCTTATGTGATGGCTATGTGT<br>R: ACCCCTTATGACGCATTCTATGT | 96 |
| *SOX2* | F: TGGACAGTTACGCGCACAT<br>R: CGAGTAGGACATGCTGTAGGT | 215 |
| *VEGFR2* | F: ACTGTCATCCTTACCAATCC<br>R: CTTCCAACTGCCAATACC | 202 |
| *VEGFR3* | F: TGCACGAGGTACATGCCAAC<br>R: GCTGCTCAAAGTCTCTCACGAA | 120 |
| *WNT4* | F: AGGAGGAGACGTGCGAGAAA<br>R: CGAGTCCATGACTTCCAGGT | 83 |
