# Supplementary material for: Menstrual Blood-Derived Endometrial Stem Cells’ Impact for the Treatment Perspective of Female Infertility
Source: Int J Mol Sci. 2021 Jun 24;22(13):6774. doi: 10.3390/ijms22136774 (PMC8268036; doi:10.3390/ijms22136774)
Supplement: Supplementary file 1 [file ijms-22-06774-s001.zip › Table S3.pdf]

Table S3. Antibodies used in Western blot analysis

Antibodies	Dilution	Manufacturer
HRP-conjugated secondary antibodies against mouse immunoglobulins (goat, polyclonal)	1:2000	Agilent Dako, Santa Clara, California, USA
HRP-conjugated secondary antibodies against rabbit immunoglobulins (goat, polyclonal)	1:2000	Agilent Dako, Santa Clara, California, USA
GAPDH (monoclonal, mouse, clone 6C5)	1:10 000	Abcam, Cambridge, UK
P53 (monoclonal, mouse, clone DO-1)	1:500	Santa Cruz Biotechnology, Santa Cruz, California, USA
P21 (monoclonal, mouse, clone 187)	1:1000	Abcam, Cambridge, UK
Cyclin A2 (monoclonal, mouse, clone 17B8.2)	1:10 000	Millipore, Billerica, Massachusetts, USA
BAX (monoclonal, rabbit, clone 2D2)	1:1000	Cell Signaling Technology, Danvers, Massachusetts, USA
C/EBPβ (monoclonal, mouse)	1:500	Santa Cruz Biotechnology, Santa Cruz, California, USA
Ezh2 (polyclonal, rabbit)	1:2000	Abcam, Cambridge, UK
Suz12 (monoclonal, rabbit, clone D39F6)	1:1000	Cell Signaling Technology, Danvers, Massachusetts, USA
H3K27me3 (polyclonal, rabbit)	1:500	Millipore, Billerica, Massachusetts, USA
HDAC1 (monoclonal, mouse, clone 10E2)	1:1000	Abcam, Cambridge, UK
HyperAcH4 (polyclonal, rabbit)	1:1000	Millipore, Billerica, Massachusetts, USA
Pan-Cadherin (polyclonal, rabbit)	1:1000	Santa Cruz Biotechnology, Santa Cruz, California, USA
