# Supplementary material for: Menstrual Blood-Derived Endometrial Stem Cells’ Impact for the Treatment Perspective of Female Infertility
Source: Int J Mol Sci. 2021 Jun 24;22(13):6774. doi: 10.3390/ijms22136774 (PMC8268036; doi:10.3390/ijms22136774)
Supplement: Supplementary file 1 [file ijms-22-06774-s001.zip › Table S5.pdf]

Table S5. The functions of 19 differently secreted proteins during decidualization of MenSCs characteristic for healthy donors and patients with unexplained infertility

Protein	Entry	Description	Accession	Process
APOC3	APOC3	Apolipoprotein C-III	P02656	GO:0051004 regulation of lipoprotein lipase activity; GO:0006641 triglyceride metabolic process; GO:0048259 regulation of receptor-mediated endocytosis; GO:0043062 extracellular structure organization; GO:0030100 regulation of endocytosis; hsa04979 Cholesterol metabolism; ***hsa03320 PPAR signaling pathway***
POTEE	POTEE	POTE ankyrin domain family member E	Q6S8J3	No process detected
AHSG	FETUA	Alpha-2-HS-glycoprotein	P02765	GO:0002576 platelet degranulation; GO:0030100 regulation of endocytosis; GO:0010951 negative regulation of endopeptidase activity; GO:0043687 post-translational protein modification; GO:0045055 regulated exocytosis; GO:0052548 regulation of endopeptidase activity
GSN	GELS	Gelsolin	P06396	GO:0046686 response to cadmium ion; GO:0048709 oligodendrocyte differentiation; GO:0070613 regulation of protein processing; GO:0051258 protein polymerization; GO:0033273 response to vitamin; GO:0042060 wound healing; GO:0003012 muscle system process; GO:0045055 regulated exocytosis; GO:0052548 regulation of endopeptidase activity; ***GO:0051015 actin filament binding***
SPARC - SPARC	SPRC	SPARC	P09486	***GO:0001937 negative regulation of endothelial cell proliferation;*** GO:0002576 platelet degranulation; GO:0046686 response to cadmium ion; GO:0043062 extracellular structure organization; GO:0030198 extracellular matrix organization; GO:0042060 wound healing; GO:0045055 regulated exocytosis; GO:0050840 extracellular matrix binding; GO:0005518 collagen binding
TIMP1	TIMP1	Metalloproteinase inhibitor 1	P01033	GO:0002576 platelet degranulation; GO:0022617 extracellular matrix disassembly; GO:0043062 extracellular structure organization; GO:0030198 extracellular matrix organization; GO:0042060 wound healing; GO:0030336 negative

				regulation of cell migration; GO:0010951 negative regulation of endopeptidase activity; GO:0043687 post-translational protein modification; GO:0045055 regulated exocytosis; GO:0052548 regulation of endopeptidase activity
APOH	APOH	Beta-2-glycoprotein 1	P02749	GO:0051004 regulation of lipoprotein lipase activity; GO:0001937 negative regulation of endothelial cell proliferation; GO:0030195 negative regulation of blood coagulation; GO:0090303 positive regulation of wound healing; GO:0002576 platelet degranulation; GO:0006641 triglyceride metabolic process; GO:0042060 wound healing; GO:0030336 negative regulation of cell migration; GO:0007596 blood coagulation; GO:0045055 regulated exocytosis; hsa04979 Cholesterol metabolism
HIST1H2BB	H2B1B	Histone H2B type 1-B	P33778	No process detected
VTN	VTNC	Vitronectin	P04004	GO:0030195 negative regulation of blood coagulation; GO:0090303 positive regulation of wound healing; ***GO:0030449 regulation of complement activation***; GO:2000257 regulation of protein activation cascade; GO:0048709 oligodendrocyte differentiation; GO:0070613 regulation of protein processing; GO:0048259 regulation of receptor-mediated endocytosis; GO:0051258 protein polymerization; GO:0033273 response to vitamin; GO:0002673 regulation of acute inflammatory response; GO:0043062 extracellular structure organization; GO:0030198 extracellular matrix organization; GO:0030100 regulation of endocytosis; GO:0010951 negative regulation of endopeptidase activity; GO:0052548 regulation of endopeptidase activity; GO:0050840 extracellular matrix binding; GO:0005518 collagen binding; ***hsa04610 Complement and coagulation cascades***
MMP1	MMP1	Interstitial collagenase	P03956	GO:0022617 extracellular matrix disassembly; GO:0043062 extracellular structure organization; GO:0030198 extracellular matrix organization; ***hsa03320 PPAR***

				signaling pathway; hsa04926 Relaxin signaling pathway
CPN1	CBPN	Carboxypeptidase N catalytic chain	P15169	***GO:0030449 regulation of complement activation***; GO:2000257 regulation of protein activation cascade; GO:0070613 regulation of protein processing; GO:0002673 regulation of acute inflammatory response
PSMB1	PSB1	Proteasome subunit beta type-1	P20618	GO:0043687 post-translational protein modification; GO:0045055 regulated exocytosis
F5	FA5	Coagulation factor V	P12259	GO:0002576 platelet degranulation; GO:0042060 wound healing; GO:0043687 post-translational protein modification; GO:0007596 blood coagulation; GO:0045055 regulated exocytosis; hsa04610 Complement and coagulation cascades
INADL	INADL	InaD-like protein	Q8NI35	***hsa04530 Tight junction***
CO3A1	CO3A1	Collagen alpha-1(III) chain	P02461	GO:0043062 extracellular structure organization; GO:0030198 extracellular matrix organization; GO:0042060 wound healing; GO:0030336 negative regulation of cell migration; GO:0007596 blood coagulation; ***hsa04926 Relaxin signaling pathway***
TKT	TKT	Transketolase	P29401	No process detected
KRT77	K2C1B	Keratin, type II cytoskeletal 1b	Q7Z794	No process detected
MYH4	MYH4	Myosin-4	Q9Y623	GO:0003012 muscle system process; ***GO:0051015 actin filament binding; hsa04530 Tight junction***
MYH1	MYH1	Myosin-1	P12882	GO:0003012 muscle system process; ***GO:0051015 actin filament binding; hsa04530 Tight junction***
