# Supplementary material for: Menstrual Blood-Derived Endometrial Stem Cells’ Impact for the Treatment Perspective of Female Infertility
Source: Int J Mol Sci. 2021 Jun 24;22(13):6774. doi: 10.3390/ijms22136774 (PMC8268036; doi:10.3390/ijms22136774)
Supplement: Supplementary file 1 [file ijms-22-06774-s001.zip › Table S6.pdf]

Table S6. Proteins detected in the secretion profile analysis of MenSCs. C - protein secreted by control MenSCs, 6d - proteins secreted by decidualized MenSCs

Process	Protein	Healthy donors	Patients with unexplained infertility
		Ratio 6d/C	Ratio 6d/C
GO:0007566 embryo implantation, GO:0007565 female pregnancy	IGFBP7	6.36	10.35
	IGFBP4	2.32	5.42
	FBLN1	0.665	0.72
	Timp1	0.455	1.14
	Timp2	3.135	5.57
	FSTL1	2.29	2.22
	PZP	11.98	26.61
	Vim	0.77	0.85
GO:0031532 actin cytoskeleton reorganization	GSN	0.455	2.25
	Actn1	0.15	0.11
	Fn1	2.3	1.39
	Myh9	0.61	0.86
	Vcl	0.395	0.2
	Ezr	0.015	0.06
hsa04610 Complement and coagulation cascades	Fga	0.12	0.14
	F10	4.85	1.13
	Serpine1	0.94	0.4
	Serpinf2	-	0.19
	CFI	0.335	0.32
	Plg	0.52	0.15
	C8b	0.23	0.49
	F5	0.56	2.6
	C3	0.2	0.29
	C4a	0.16	0.18
	Vtn	0.85	1.05
	C5	0.1	0.13
	C7	1.68	1.88
	A2M	0.08	0.12

Process	Protein	Healthy donors	Patients with unexplained infertility
		Ratio 6d/C	Ratio 6d/C
hsa04915 Estrogen signaling pathway	HspA8	0.165	0.24
	Hsp90aa1	0.24	0.18
	Hspa1l	0.13	0.07
	Mmp2	1.975	2.22
	Krt9	1.44	2.09
hsa04926 Relaxin signaling pathway	Col3a1	0.685	1.3
	Col1a2	7.23	5.27
	Col4a2	1.87	2.15
	Mmp1	-	1.775
hsa04540 Gap junction, hsa04530 Tight junction	Actn4	-	0.073
	Tuba1c	0.115	0.17
	Tubb1	0.115	0.17
hsa00010 Glycolysis/ Gluconeogenesis	Ldha	-	0.09
	Pkm	0.07	0.19
	Eno2	-	0.19
	Aldoa	1.02	1.08
	GAPDH	2.09	2.33
GO:0016525 negative regulation of angiogenesis	Thbs2	6.8	3.97
	Thbs1	3.6	1.32
hsa04512 ECM-receptor interaction	Col6a1	0.66	0.66
	Col6a3	0.395	0.86
	Actn1	0.15	0.11
	Flna	0.045	0.64
	Tln1	0.07	0.10
